# Bis(2,2′-bipyridine-κ^2^ *N*,*N*′)tris­(nitrato-κ^2^ *O*,*O*′)erbium(III)

**DOI:** 10.1107/S1600536812014031

**Published:** 2012-04-13

**Authors:** Hua Yang

**Affiliations:** a College of Chemistry and Chemical Engineering, Yan’an University, Yan’an, Shaanxi 716000, People’s Republic of China

## Abstract

The asymmetric unit of the title compound, [Er(NO_3_)_3_(C_10_H_8_N_2_)_2_], contains one-half mol­ecule situated on a twofold rotation axis. The Er^III^ ion is in a tenfold coordination by six O atoms from three NO_3_^−^ anions and four N atoms from two 2,2′-bipyridine ligands in a distorted bicapped dodeca­hedral geometry. In the crystal, weak C—H⋯O hydrogen bonds hold the mol­ecules together.

## Related literature

For the crystal structures of related erbium complexes with 2,2′-bipyridine, see: Lu *et al.* (1995 ▶); Su *et al.* (1996 ▶); Staveren *et al.* (2000 ▶); Roh *et al.* (2005 ▶); Estrader *et al.* (2006 ▶); Ren *et al.* (2006 ▶). For potential applications of related complexes, see: Huskowska *et al.* (2002 ▶); Li *et al.* (2007 ▶); Puntus *et al.* (2009 ▶).
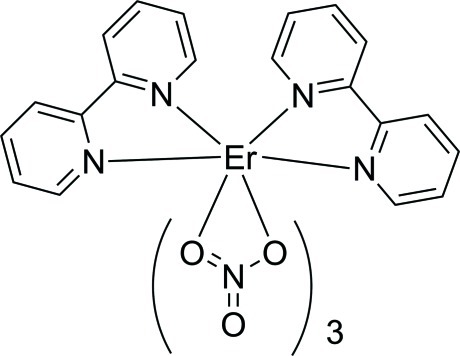


## Experimental

### 

#### Crystal data

[Er(NO_3_)_3_(C_10_H_8_N_2_)_2_]
*M* *_r_* = 665.66
Orthorhombic, 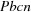

*a* = 16.5762 (4) Å
*b* = 9.1158 (2) Å
*c* = 15.0288 (4) Å
*V* = 2270.93 (10) Å^3^
*Z* = 4
Mo *K*α radiation
μ = 3.76 mm^−1^
*T* = 296 K
0.25 × 0.23 × 0.18 mm

#### Data collection

Bruker SMART CCD diffractometer
Absorption correction: multi-scan (*SADABS*; Bruker, 2000 ▶) *T*_min_ = 0.453, *T*_max_ = 0.551
11877 measured reflections
2859 independent reflections
2127 reflections with *I* > 2σ(*I*)
*R*_int_ = 0.016

#### Refinement

*R*[*F* ^2^ > 2σ(*F* ^2^)] = 0.018
*wR*(*F* ^2^) = 0.051
*S* = 1.06
2859 reflections
169 parameters
8 restraints
H-atom parameters constrained
Δρ_max_ = 0.51 e Å^−3^
Δρ_min_ = −1.07 e Å^−3^

### 

Data collection: *SMART* (Bruker, 2000 ▶); cell refinement: *SAINT* (Bruker, 2000 ▶); data reduction: *SAINT*; program(s) used to solve structure: *SHELXS97* (Sheldrick, 2008 ▶); program(s) used to refine structure: *SHELXL97* (Sheldrick, 2008 ▶); molecular graphics: *SHELXTL* (Sheldrick, 2008 ▶); software used to prepare material for publication: *SHELXTL*.

## Supplementary Material

Crystal structure: contains datablock(s) I, global. DOI: 10.1107/S1600536812014031/cv5264sup1.cif

Structure factors: contains datablock(s) I. DOI: 10.1107/S1600536812014031/cv5264Isup2.hkl

Additional supplementary materials:  crystallographic information; 3D view; checkCIF report

## Figures and Tables

**Table 1:** Hydrogen-bond geometry (Å, °)

*D*—H⋯*A*	*D*—H	H⋯*A*	*D*⋯*A*	*D*—H⋯*A*
C5—H5⋯O6^i^	0.93	2.45	3.325 (6)	157
C7—H7⋯O4^ii^	0.93	2.49	3.274 (6)	142

Symmetry codes: (i) 
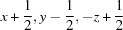
; (ii) 
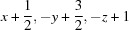
.

**Fig. 1.**
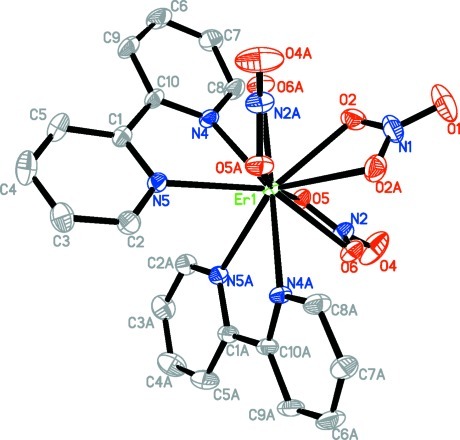
The molecular structure of the title compound, with atom labels and 30% probability displacement ellipsoids [symmetry code: (A) -x, y, -z + 1/2)]. The H atoms have been omitted for clarity.

**Fig. 2.**
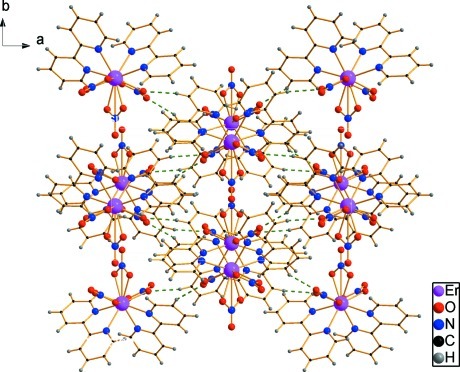
A portion of the crystal packing viewed down the c-axis. Hydrogen bonds are shown as dashed lines.

## supplementary crystallographic information

### Comment

The synthesis and characterization of lanthanide complexes supported by *N*,*O*-chelating ligands and 2,2'-bipyridine have attracted continuous interest, due to the potential application of these compounds in magnetic, electronic and luminescent devices (Puntus *et al.*, 2009). Particularly, it was found that the coordination of 2,2'-bipyridine to the metal centers of the complexes could effectively tune the structures and properties of the resulting complexes (Li *et al.*, 2007; Huskowska *et al.*, 2002). Several erbium complexes with 2,2'-bipyridine have been reported (Staveren *et al.*, 2000; Su *et al.*, 1996; Lu *et al.*, 1995; Estrader *et al.*, 2006; Ren *et al.*, 2006; Roh *et al.*, 2005). In our attempts to synthesize the Er^III^ complex supported by salicylaldehyde thiosemicarbazone and 2,2'-bipyridine, we obtained the title compound (I).

In (I) (Fig. 1), each NO_3_^-^ anion chelates to the metal center in a bidentate fashion. The central Er^III^ ion adopts distorted bicapped dodecahedron geometry. The weak intermolecular C—H···O hydrogen bonds (Table 1) held the molecules together (Fig. 2).

### Experimental

A mixture of Er(NO_3_)_3_.6H_2_O (0.0460 g, 0.1 mmol), 2,2'-bipyridine (0.0312 g, 0.2 mmol), salicylaldehyde thiosemicarbazone (0.0195 g, 0.1 mmol) and C_2_H_5_OH (3 ml) was sealed in a 6 ml Pyrex-tube. The tube was heated at 70 *^o^*C for 3 days under autogenous pressure. Cooling of the resultant solution to room temperature gave light pink crystals. The crystals were collected by filtration, washed with C_2_H_5_OH (2 ml) and dried in air.

### Refinement

H atoms were placed in calculated positions with C—H = 0.93 (aromatic and pyrrole) and refined in riding mode, with *U*_iso_(H) = 1.2 *U*_eq_(C).

### Crystal data

**Table:**

[Er(NO_3_)_3_(C_10_H_8_N_2_)_2_]	*F*(000) = 1300
*M**_r_* = 665.66	*D*_x_ = 1.947 Mg m^−^^3^
Orthorhombic, *P**b**c**n*	Mo *K*α radiation, λ = 0.71073 Å
Hall symbol: -P 2n 2ab	Cell parameters from 5852 reflections
*a* = 16.5762 (4) Å	θ = 2.6–26.5°
*b* = 9.1158 (2) Å	µ = 3.76 mm^−^^1^
*c* = 15.0288 (4) Å	*T* = 296 K
*V* = 2270.93 (10) Å^3^	Block, pink
*Z* = 4	0.25 × 0.23 × 0.18 mm

### Data collection

**Table:**

Bruker SMART CCD diffractometer	2859 independent reflections
Radiation source: fine-focus sealed tube	2127 reflections with *I* > 2σ(*I*)
Graphite monochromator	*R*_int_ = 0.016
φ scans and ω scans	θ_max_ = 28.5°, θ_min_ = 2.5°
Absorption correction: multi-scan (*SADABS*; Bruker, 2000)	*h* = −22→22
*T*_min_ = 0.453, *T*_max_ = 0.551	*k* = −12→7
11877 measured reflections	*l* = −20→15

### Refinement

**Table:**

Refinement on *F*^2^	Primary atom site location: structure-invariant direct methods
Least-squares matrix: full	Secondary atom site location: difference Fourier map
*R*[*F*^2^ > 2σ(*F*^2^)] = 0.018	Hydrogen site location: inferred from neighbouring sites
*wR*(*F*^2^) = 0.051	H-atom parameters constrained
*S* = 1.06	*w* = 1/[σ^2^(*F*_o_^2^) + (0.0189*P*)^2^ + 3.3713*P*] where *P* = (*F*_o_^2^ + 2*F*_c_^2^)/3
2859 reflections	(Δ/σ)_max_ = 0.010
169 parameters	Δρ_max_ = 0.51 e Å^−^^3^
8 restraints	Δρ_min_ = −1.07 e Å^−^^3^

### Special details

**Table:**

Geometry. All e.s.d.'s (except the e.s.d. in the dihedral angle between two l.s. planes) are estimated using the full covariance matrix. The cell e.s.d.'s are taken into account individually in the estimation of e.s.d.'s in distances, angles and torsion angles; correlations between e.s.d.'s in cell parameters are only used when they are defined by crystal symmetry. An approximate (isotropic) treatment of cell e.s.d.'s is used for estimating e.s.d.'s involving l.s. planes.
Refinement. Refinement of *F*^2^ against ALL reflections. The weighted *R*-factor *wR* and goodness of fit *S* are based on *F*^2^, conventional *R*-factors *R* are based on *F*, with *F* set to zero for negative *F*^2^. The threshold expression of *F*^2^ > σ(*F*^2^) is used only for calculating *R*-factors(gt) *etc*. and is not relevant to the choice of reflections for refinement. *R*-factors based on *F*^2^ are statistically about twice as large as those based on *F*, and *R*- factors based on ALL data will be even larger.

### Fractional atomic coordinates and isotropic or equivalent isotropic displacement parameters (Å^2^)

**Table:**

	*x*	*y*	*z*	*U*_iso_*/*U*_eq_	
Er1	0.0000	0.59232 (2)	0.2500	0.02279 (8)	
O1	0.0000	1.0374 (6)	0.2500	0.0790 (13)	
O2	0.0381 (2)	0.8323 (4)	0.3082 (3)	0.0487 (8)	
O4	−0.1240 (3)	0.7247 (6)	0.4738 (3)	0.0789 (15)	
O5	−0.01985 (19)	0.6182 (4)	0.4172 (2)	0.0391 (8)	
O6	−0.11602 (17)	0.6865 (4)	0.3319 (2)	0.0393 (7)	
N1	0.0000	0.9058 (6)	0.2500	0.0535 (12)	
N2	−0.0876 (2)	0.6777 (5)	0.4101 (3)	0.0411 (9)	
N4	0.12765 (19)	0.5175 (4)	0.3252 (2)	0.0305 (8)	
N5	0.0672 (2)	0.3730 (4)	0.1854 (2)	0.0295 (7)	
C1	0.1431 (2)	0.3387 (5)	0.2090 (3)	0.0320 (9)	
C2	0.0322 (3)	0.2940 (5)	0.1210 (3)	0.0386 (10)	
H2	−0.0203	0.3175	0.1044	0.046*	
C3	0.0699 (3)	0.1800 (6)	0.0783 (4)	0.0487 (12)	
H3	0.0435	0.1277	0.0338	0.058*	
C4	0.1470 (4)	0.1449 (7)	0.1025 (4)	0.0591 (15)	
H4	0.1738	0.0676	0.0750	0.071*	
C5	0.1845 (3)	0.2258 (6)	0.1683 (4)	0.0495 (13)	
H5	0.2372	0.2043	0.1851	0.059*	
C6	0.2890 (3)	0.5056 (6)	0.3713 (4)	0.0508 (13)	
H6	0.3436	0.5042	0.3852	0.061*	
C7	0.2369 (3)	0.5918 (6)	0.4189 (4)	0.0466 (13)	
H7	0.2549	0.6468	0.4671	0.056*	
C8	0.1569 (3)	0.5945 (5)	0.3933 (3)	0.0401 (11)	
H8	0.1215	0.6534	0.4254	0.048*	
C9	0.2597 (3)	0.4212 (6)	0.3027 (4)	0.0430 (12)	
H9	0.2941	0.3599	0.2709	0.052*	
C10	0.1788 (2)	0.4283 (5)	0.2813 (3)	0.0312 (9)	

### Atomic displacement parameters (Å^2^)

**Table:**

	*U*^11^	*U*^22^	*U*^33^	*U*^12^	*U*^13^	*U*^23^
Er1	0.01451 (11)	0.02538 (13)	0.02849 (13)	0.000	−0.00210 (10)	0.000
O1	0.130 (4)	0.030 (2)	0.077 (3)	0.000	0.029 (2)	0.000
O2	0.0448 (19)	0.0446 (19)	0.057 (2)	−0.0123 (16)	0.0024 (16)	−0.0140 (16)
O4	0.061 (3)	0.131 (4)	0.045 (2)	0.038 (3)	0.012 (2)	−0.023 (3)
O5	0.0302 (15)	0.0477 (19)	0.0395 (18)	0.0095 (13)	−0.0062 (13)	−0.0049 (15)
O6	0.0268 (15)	0.052 (2)	0.0386 (18)	0.0071 (14)	−0.0040 (13)	−0.0030 (15)
N1	0.068 (4)	0.027 (2)	0.066 (3)	0.000	0.027 (2)	0.000
N2	0.0324 (19)	0.052 (2)	0.039 (2)	0.0068 (18)	0.0021 (17)	−0.0051 (19)
N4	0.0216 (15)	0.0360 (19)	0.034 (2)	0.0031 (14)	−0.0041 (14)	−0.0003 (16)
N5	0.0270 (17)	0.0315 (18)	0.0299 (18)	0.0010 (14)	0.0023 (14)	−0.0008 (15)
C1	0.029 (2)	0.034 (2)	0.033 (2)	0.0056 (17)	0.0025 (18)	0.0036 (19)
C2	0.038 (2)	0.040 (2)	0.037 (2)	0.000 (2)	−0.001 (2)	−0.002 (2)
C3	0.062 (3)	0.044 (3)	0.040 (3)	0.002 (2)	0.001 (2)	−0.011 (2)
C4	0.071 (4)	0.053 (3)	0.053 (4)	0.022 (3)	0.007 (3)	−0.014 (3)
C5	0.042 (3)	0.056 (3)	0.051 (3)	0.020 (2)	0.005 (2)	−0.003 (3)
C6	0.024 (2)	0.065 (3)	0.064 (4)	0.004 (2)	−0.013 (2)	0.009 (3)
C7	0.036 (3)	0.052 (3)	0.051 (3)	−0.001 (2)	−0.019 (2)	−0.002 (2)
C8	0.029 (2)	0.049 (3)	0.042 (3)	0.0061 (19)	−0.0081 (19)	−0.008 (2)
C9	0.025 (2)	0.051 (3)	0.053 (3)	0.0123 (19)	−0.002 (2)	0.004 (2)
C10	0.0232 (19)	0.036 (2)	0.034 (2)	0.0068 (16)	0.0002 (17)	0.0059 (18)

### Geometric parameters (Å, º)

**Table:**

Er1—O2^i^	2.439 (3)	N5—C1	1.345 (5)
Er1—O2	2.439 (3)	C1—C5	1.380 (6)
Er1—O6	2.439 (3)	C1—C10	1.482 (7)
Er1—O6^i^	2.439 (3)	C2—C3	1.372 (7)
Er1—N5	2.486 (3)	C2—H2	0.9300
Er1—N5^i^	2.486 (3)	C3—C4	1.367 (8)
Er1—N4^i^	2.494 (3)	C3—H3	0.9300
Er1—N4	2.494 (3)	C4—C5	1.382 (8)
Er1—O5^i^	2.544 (3)	C4—H4	0.9300
Er1—O5	2.544 (3)	C5—H5	0.9300
O1—N1	1.199 (8)	C6—C7	1.369 (8)
O2—N1	1.270 (5)	C6—C9	1.375 (8)
O4—N2	1.210 (5)	C6—H6	0.9300
O5—N2	1.251 (5)	C7—C8	1.381 (6)
O6—N2	1.270 (5)	C7—H7	0.9300
N1—O2^i^	1.270 (5)	C8—H8	0.9300
N4—C8	1.333 (6)	C9—C10	1.381 (6)
N4—C10	1.347 (5)	C9—H9	0.9300
N5—C2	1.339 (6)		
			
O2^i^—Er1—O2	52.50 (19)	N2—O5—Er1	94.2 (3)
O2^i^—Er1—O6	70.17 (12)	N2—O6—Er1	98.8 (2)
O2—Er1—O6	73.01 (12)	O1—N1—O2^i^	121.9 (3)
O2^i^—Er1—O6^i^	73.01 (12)	O1—N1—O2	121.9 (3)
O2—Er1—O6^i^	70.17 (12)	O2^i^—N1—O2	116.2 (5)
O6—Er1—O6^i^	138.78 (17)	O4—N2—O5	122.3 (4)
O2^i^—Er1—N5	134.21 (13)	O4—N2—O6	121.7 (4)
O2—Er1—N5	138.18 (12)	O5—N2—O6	116.0 (4)
O6—Er1—N5	146.44 (11)	C8—N4—C10	117.7 (4)
O6^i^—Er1—N5	74.51 (11)	C8—N4—Er1	120.8 (3)
O2^i^—Er1—N5^i^	138.18 (12)	C10—N4—Er1	118.5 (3)
O2—Er1—N5^i^	134.21 (13)	C2—N5—C1	118.1 (4)
O6—Er1—N5^i^	74.51 (12)	C2—N5—Er1	121.4 (3)
O6^i^—Er1—N5^i^	146.44 (11)	C1—N5—Er1	120.2 (3)
N5—Er1—N5^i^	72.93 (16)	N5—C1—C5	121.5 (4)
O2^i^—Er1—N4^i^	82.13 (12)	N5—C1—C10	116.0 (4)
O2—Er1—N4^i^	128.87 (12)	C5—C1—C10	122.5 (4)
O6—Er1—N4^i^	69.87 (11)	N5—C2—C3	123.3 (5)
O6^i^—Er1—N4^i^	122.46 (11)	N5—C2—H2	118.4
N5—Er1—N4^i^	89.02 (11)	C3—C2—H2	118.4
N5^i^—Er1—N4^i^	64.99 (12)	C4—C3—C2	118.6 (5)
O2^i^—Er1—N4	128.87 (12)	C4—C3—H3	120.7
O2—Er1—N4	82.13 (12)	C2—C3—H3	120.7
O6—Er1—N4	122.46 (11)	C3—C4—C5	119.1 (5)
O6^i^—Er1—N4	69.87 (11)	C3—C4—H4	120.4
N5—Er1—N4	64.99 (12)	C5—C4—H4	120.4
N5^i^—Er1—N4	89.02 (11)	C1—C5—C4	119.4 (5)
N4^i^—Er1—N4	148.26 (17)	C1—C5—H5	120.3
O2^i^—Er1—O5^i^	66.21 (12)	C4—C5—H5	120.3
O2—Er1—O5^i^	103.72 (12)	C7—C6—C9	119.4 (4)
O6—Er1—O5^i^	124.56 (10)	C7—C6—H6	120.3
O6^i^—Er1—O5^i^	50.76 (10)	C9—C6—H6	120.3
N5—Er1—O5^i^	68.33 (11)	C6—C7—C8	118.1 (5)
N5^i^—Er1—O5^i^	121.21 (11)	C6—C7—H7	121.0
N4^i^—Er1—O5^i^	71.80 (11)	C8—C7—H7	121.0
N4—Er1—O5^i^	111.28 (11)	N4—C8—C7	123.6 (5)
O2^i^—Er1—O5	103.72 (12)	N4—C8—H8	118.2
O2—Er1—O5	66.21 (12)	C7—C8—H8	118.2
O6—Er1—O5	50.76 (10)	C6—C9—C10	119.4 (5)
O6^i^—Er1—O5	124.56 (10)	C6—C9—H9	120.3
N5—Er1—O5	121.21 (11)	C10—C9—H9	120.3
N5^i^—Er1—O5	68.33 (11)	N4—C10—C9	121.7 (5)
N4^i^—Er1—O5	111.28 (11)	N4—C10—C1	116.1 (4)
N4—Er1—O5	71.80 (11)	C9—C10—C1	122.2 (4)
O5^i^—Er1—O5	169.37 (15)		
			
O2^i^—Er1—O2—N1	0.001 (1)	O5^i^—Er1—N4—C10	−33.6 (3)
O6—Er1—O2—N1	−77.72 (18)	O5—Er1—N4—C10	157.2 (3)
O6^i^—Er1—O2—N1	83.41 (19)	N1—Er1—N4—C10	−124.0 (3)
N5—Er1—O2—N1	117.4 (2)	O2^i^—Er1—N5—C2	−65.7 (4)
N5^i^—Er1—O2—N1	−124.34 (19)	O2—Er1—N5—C2	−144.9 (3)
N4^i^—Er1—O2—N1	−32.8 (3)	O6—Er1—N5—C2	61.9 (4)
N4—Er1—O2—N1	154.8 (2)	O6^i^—Er1—N5—C2	−111.8 (4)
O5^i^—Er1—O2—N1	44.7 (2)	N5^i^—Er1—N5—C2	76.4 (3)
O5—Er1—O2—N1	−131.7 (2)	N4^i^—Er1—N5—C2	12.3 (3)
O2^i^—Er1—O5—N2	46.4 (3)	N4—Er1—N5—C2	173.5 (4)
O2—Er1—O5—N2	83.9 (3)	O5^i^—Er1—N5—C2	−58.4 (3)
O6—Er1—O5—N2	−3.1 (2)	O5—Er1—N5—C2	126.8 (3)
O6^i^—Er1—O5—N2	125.0 (3)	N1—Er1—N5—C2	−103.6 (3)
N5—Er1—O5—N2	−142.8 (3)	O2^i^—Er1—N5—C1	108.3 (3)
N5^i^—Er1—O5—N2	−90.4 (3)	O2—Er1—N5—C1	29.1 (4)
N4^i^—Er1—O5—N2	−40.4 (3)	O6—Er1—N5—C1	−124.1 (3)
N4—Er1—O5—N2	173.2 (3)	O6^i^—Er1—N5—C1	62.2 (3)
O5^i^—Er1—O5—N2	64.6 (3)	N5^i^—Er1—N5—C1	−109.6 (4)
O2^i^—Er1—O6—N2	−125.3 (3)	N4^i^—Er1—N5—C1	−173.7 (3)
O2—Er1—O6—N2	−69.8 (3)	N4—Er1—N5—C1	−12.5 (3)
O6^i^—Er1—O6—N2	−97.3 (3)	O5^i^—Er1—N5—C1	115.6 (3)
N5—Er1—O6—N2	91.9 (3)	O5—Er1—N5—C1	−59.2 (3)
N5^i^—Er1—O6—N2	77.5 (3)	N1—Er1—N5—C1	70.4 (4)
N4^i^—Er1—O6—N2	146.1 (3)	C2—N5—C1—C5	0.2 (7)
N4—Er1—O6—N2	−1.1 (3)	Er1—N5—C1—C5	−174.0 (4)
O5^i^—Er1—O6—N2	−165.0 (3)	C2—N5—C1—C10	−179.3 (4)
O5—Er1—O6—N2	3.1 (2)	Er1—N5—C1—C10	6.5 (5)
Er1—O2—N1—O1	180.000 (1)	C1—N5—C2—C3	0.1 (7)
Er1—O2—N1—O2^i^	−0.001 (1)	Er1—N5—C2—C3	174.3 (4)
Er1—O5—N2—O4	−174.6 (5)	N5—C2—C3—C4	0.0 (8)
Er1—O5—N2—O6	5.1 (4)	C2—C3—C4—C5	−0.5 (9)
Er1—O6—N2—O4	174.4 (5)	N5—C1—C5—C4	−0.7 (8)
Er1—O6—N2—O5	−5.4 (4)	C10—C1—C5—C4	178.8 (5)
O2^i^—Er1—N4—C8	50.6 (4)	C3—C4—C5—C1	0.9 (9)
O2—Er1—N4—C8	24.8 (4)	C9—C6—C7—C8	−2.5 (8)
O6—Er1—N4—C8	−39.2 (4)	C10—N4—C8—C7	2.5 (7)
O6^i^—Er1—N4—C8	96.5 (4)	Er1—N4—C8—C7	−157.8 (4)
N5—Er1—N4—C8	178.3 (4)	C6—C7—C8—N4	0.3 (8)
N5^i^—Er1—N4—C8	−110.1 (4)	C7—C6—C9—C10	2.0 (8)
N4^i^—Er1—N4—C8	−143.9 (4)	C8—N4—C10—C9	−3.1 (7)
O5^i^—Er1—N4—C8	126.5 (3)	Er1—N4—C10—C9	157.7 (4)
O5—Er1—N4—C8	−42.6 (3)	C8—N4—C10—C1	176.9 (4)
N1—Er1—N4—C8	36.1 (4)	Er1—N4—C10—C1	−22.3 (5)
O2^i^—Er1—N4—C10	−109.6 (3)	C6—C9—C10—N4	0.9 (8)
O2—Er1—N4—C10	−135.3 (3)	C6—C9—C10—C1	−179.1 (5)
O6—Er1—N4—C10	160.6 (3)	N5—C1—C10—N4	10.4 (6)
O6^i^—Er1—N4—C10	−63.6 (3)	C5—C1—C10—N4	−169.1 (4)
N5—Er1—N4—C10	18.2 (3)	N5—C1—C10—C9	−169.6 (4)
N5^i^—Er1—N4—C10	89.7 (3)	C5—C1—C10—C9	11.0 (7)
N4^i^—Er1—N4—C10	56.0 (3)		

Symmetry code: (i) −*x*, *y*, −*z*+1/2.

### Hydrogen-bond geometry (Å, º)

**Table:**

*D*—H···*A*	*D*—H	H···*A*	*D*···*A*	*D*—H···*A*
C5—H5···O6^ii^	0.93	2.45	3.325 (6)	157
C7—H7···O4^iii^	0.93	2.49	3.274 (6)	142

Symmetry codes: (ii) *x*+1/2, *y*−1/2, −*z*+1/2; (iii) *x*+1/2, −*y*+3/2, −*z*+1.
